# The ICF as a common language for rehabilitation goal-setting: comparing client and professional priorities

**DOI:** 10.1186/1477-7525-9-87

**Published:** 2011-10-07

**Authors:** Michal Harty, Maryka Griesel, Aletia van der Merwe

**Affiliations:** 1 Centre for Augmentative and Alternative Communication, University of Pretoria, Corner of Lynwood and Roper Streets, Pretoria, 0028, South Africa

## Abstract

**Background:**

Joint rehabilitation goals are an important component for effective teamwork in the rehabilitation field. The activities and participation domain of the ICF provides a common language for professionals when setting these goals. Involving clients in the formulation of rehabilitation goals is gaining momentum as part of a person-centred approach to rehabilitation. However, this is particularly difficult when clients have an acquired communication disability. The expressive communication difficulties negatively affect the consensus building process. As a result, obtaining information regarding rehabilitation goals from professionals and their clients warrants further investigation for this particular population.

**Methods:**

This comparative study investigated clients and their assigned rehabilitation professionals' perception of the importance of ICF activities and participation domains for inclusion in their rehabilitation program. Twelve clients in an acute rehabilitation centre and twenty of their corresponding rehabilitation professionals participated in an activity using the Talking Mats™ visual framework for goal setting. Each participant rated the importance of the nine activities and participation domains of the ICF for inclusion in their current rehabilitation program.

**Results:**

The ICF domains which consistently appear as very important across these groups are mobility, self-care and communication. Domains which consistently appear in the lower third of the rankings include spare time, learning and thinking and domestic life. Results indicate however that no statistical significant differences exist in terms of the individual domains across each of the participant groups. Within group differences however indicated that amongst the speech-language therapists and physiotherapists there was a statistical significant difference between spare time activities and communication and mobility.

**Conclusions:**

Findings indicate that consensus is possible amongst professionals and clients even within an acute-rehabilitation setting. In addition, the Talking Mats™ visual framework appears to be a valid protocol for including clients with acquired communication disabilities in the process of obtaining consensus during goal-setting.

## Background

The concept of person-centred care is currently receiving attention in the rehabilitation literature [1]. Person or client-centred care implies adherence to at least 3 principles namely: the client themselves are at the centre of the care process [2]; the clients autonomy is the starting point of the rehabilitation [3] and the relationship with professional service providers is characterized by a power through relationship not a power over relationship [4]. This implies building partnerships with clients and their families in which they are valued members of the rehabilitation team [4]. Building authentic partnerships with clients has distinct implications for the rehabilitation process in general, and in particular, on who decides on which goals will be addressed during the rehabilitation process. Reaching consensus with all the team members involved in establishing the rehabilitation goals is dependent on active participation by all the team members. Active participation increases the likelihood of positive and sustainable outcomes [5], as everyone is in agreement as to which goals are particularly important for a specific client.

Establishing consensus is contingent upon establishing a common language which all members of the rehabilitation team (including the clients themselves) can use when describing health and health-related constructs [6]. Person-centred care advocates for understanding the client and their level of functioning and participation in relation to activities within their environment [7]. In recent years, there has been an increased interest in participation in socially-valued activities as an outcome of rehabilitation. Rehabilitation professionals therefore need to evaluate clients' functioning in everyday activities, in order to identify existing barriers and facilitate optimal participation within these activities. From a rehabilitation perspective, the International Classification of Functioning, Disability and Health (ICF), as a bio-psychosocial framework, can be used to describe the functioning of individuals with disabilities within and across different contexts [6]. The ICF distinguishes different components which operationalize the interplay between the person, their ability and the environment. These include components of individual functioning (body structures, body functions; activities and participation) and contextual components (environmental factors and personal factors) [7]. Furthermore, the ICF delineates nine domains under the activities and participation component which can be used to facilitate the description of how disability impacts on an individual's level of functioning within certain major life activities. Although the ICF depicts activities and participation separately in their graphic representation, it uses one coding structure for both activity and participation, as participation occurs across all the major life activities of an individual [7]. Difficulties at this level are described as activity limitations or participation restrictions. There has been some discussion relating to the definition of, and distinction between, activity and participation as defined within the nine activities and participation domains. Certain authors state that a lack of conceptual clarity exists between the concept of activity and the concept of participation as operationalized within the ICF, and argue that certain of the domains are more likely to operationalize activity and others participation [7].

In spite of this debate, component of the ICF have been successfully used to conceptualize meaningful participation in activities across numerous aetiologies and types of disability namely chronic disability [8], children with disabilities [9], physical disability [10], and severe disability [11]. A need remains, however, for further documentation regarding the client's own perception of rehabilitation priorities. This is particularly true for individuals who have an acquired expressive communication disorder. These individuals would benefit substantially from person-centred care as it is exactly their autonomy which has been affected by the onset of their disability [5]. As adults, it is highly likely that they would have been involved in many socially valued adult roles, and been independent contributing members of society. Subsequent to the onset of the acquired disability, many of these individuals are now unable to communicate with unfamiliar communication partners using their natural voice; may have difficulties in maintaining attention and focus over extended periods of time; and may have additional concomitant physical limitations. The presence of one or more of these factors renders it difficult for them to contribute in a meaningful manner when the goals for their rehabilitation are being constructed.

In recent years, however, tools have been developed which attempt to translate elements of the ICF framework for use within rehabilitation settings which subscribe to the ethos of person-centred care, and client involvement in the process of goal-setting. Talking Mats™ [12] is one such example. Talking Mats™ is a visual communication framework which uses pictures symbols as the basis for communication [12]. In the Talking Mats™ package, items from the ICF, are depicted in picture format using the commercially available picture communication symbols (PCS). These pictorial representations of complex rehabilitation issues, enables clients with communication difficulties to actively participate in the goal-setting process. They use the graphic representation of the items to facilitate their understanding of, and active participation in, the goal-setting process" [13-15]. This technique has been successfully used to solicit input regarding rehabilitation priorities from various adult populations with acquired communication disorders such as stroke, traumatic brain injury and Alzheimer's disease [13-15]. Studies employing this type of methodology indicate some degree of overlap in the rehabilitation goal-setting priorities highlighted by the professionals and those highlighted by the clients and family members themselves [16].

To date, however, there is a paucity of published data that determines if consensus is possible between rehabilitation professionals and clients in an acute-rehabilitation centre. The acute stage of rehabilitation is a particularly important stage in the rehabilitation process as it is the first opportunity for rehabilitation professionals to develop authentic partnerships with these clients. Due to the length of time that many of these clients will be accessing rehabilitation services, it is crucial to employ person-centred care principles in the rehabilitation process in an attempt to ensure that client remains engaged in the rehabilitation process. Therefore in the present study clients and professionals at an acute-rehabilitation centre were asked to rate the importance of the nine ICF activities and participation domains in terms of their inclusion as a current rehabilitation goal, using the Talking Mats™ visual framework. This was viewed as an initial step in establishing rehabilitation goals which reflect the priorities of all team members.

## Methods

### Research design and ethical considerations

**Figure 1:**
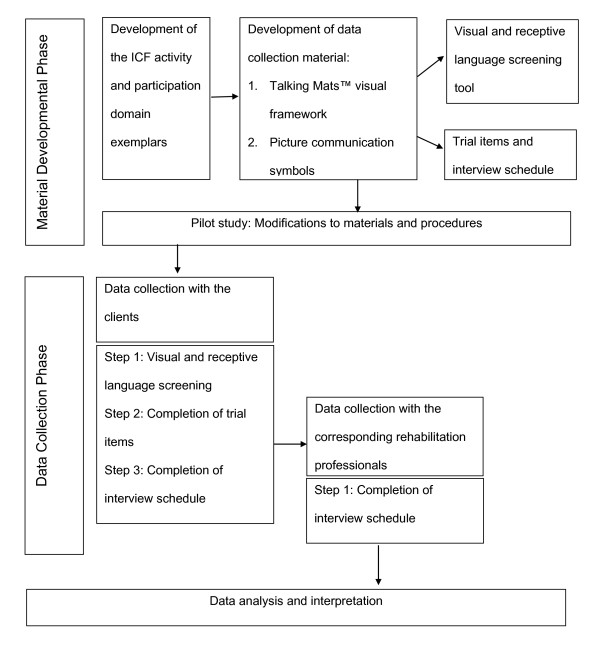
**Schematic representation of the research process**.

This study uses a comparative design [17]. An adapted structured interview format, namely the Talking Mats™ visual framework [13], was to used to capture both professional and clients ratings of the importance of the nine ICF activities and participation domains in the current rehabilitation program. Figure 1 provides a schematic representation of the research process.

Ethical clearance was granted by the University of Pretoria and the research adhered to ethical guidelines stipulated by the University. Written consent was then obtained from the selected rehabilitation centre and all the candidates who met the selection criteria were invited to participate in the study. Participants were informed that their participation in the study was voluntary and that they may withdraw at any stage during the duration of the study without any negative consequences to their current rehabilitation program. Participants gave consent for the sessions to be recorded. These video recordings were used to establish procedural integrity.

### Sampling and participant selection

#### Description of the research site - the rehabilitation centre

The rehabilitation centre which was the site for main data collection was purposefully selected and is funded by local government, has been in existence for longer than 5 years and serves individuals from a relatively large ethno-graphically diverse geographic region of the Western Cape Province of South Africa. Purposeful sampling was adopted in order to ensure that the site contained clients who had an acquired communication disability. All clients presented with an acquired communication disorder e.g. expressive aphasia, apraxia or dysarthria as a result of either traumatic brain injury (TBI) or stroke and were currently receiving rehabilitation services at the chosen rehabilitation centre. This ensured that the clients would provide relevant data which would assist with answering the aims of the research questions during the data collection procedures [17]. All the clients within the rehabilitation centre who met the selection criteria and consented to participate were included in the study. The rehabilitation professionals worked at the chosen rehabilitation centre on a permanent basis and were directly involved in decisions regarding the clients' rehabilitation. The pilot study was conducted at two rehabilitation centres in a different geographical area to the centre identified for the main study. The same procedures and selection criteria were employed in order to select participants for the pilot study.

#### Description of participants

**Figure 2:**
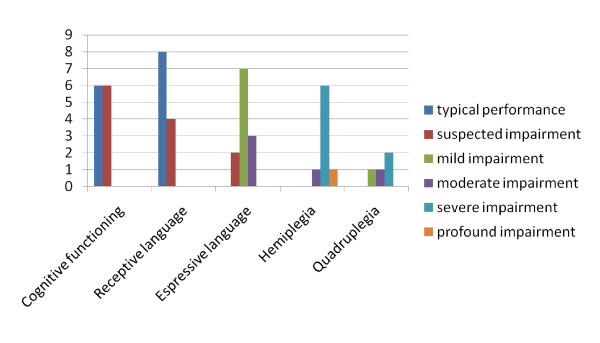
**Functioning of clients according to the Abilities Index **[18].

Twelve clients and the 20 rehabilitation professionals treating them (including occupational therapists, physiotherapists, speech-language therapists and social workers) participated in the study. The professionals described each client in terms of the clients' current motor, cognitive and communication skills using the Abilities Index [18]. Figure 2 demonstrates the number of participants whose functioning fell within each of the predetermined levels of impairment.

All of the clients had some degree of motor involvement. The majority (n = 7) had mild expressive language disorder, but nevertheless passed the receptive language screening test. Three participants provided consent for the study, but did not pass the receptive language screening test and were therefore excluded from the study. Of the remaining clients five were male and seven were female. Seven of the clients were between 25-40 years of age, three were above 40 years of age and two were below 25 yrs of age. Seven of the clients were diagnosed as having had a stroke while five clients were diagnosed with a traumatic brain injury.

The 20 professionals who participated consisted of six occupational therapists, seven physiotherapists, two speech-language therapists and five social workers. Half of professional participants (n = 10) had been employed in the field of rehabilitation for longer than five years and half (n = 10) had between one and five years of experience in the rehabilitation field. The majority (n = 15) of professionals, however, indicated that they felt very comfortable in discussing goal-setting priorities with clients. Nineteen of the 20 professionals were female.

### Material Development

For the purpose of this research certain material was developed. The exemplars of the ICF activities and participation domains were developed (and can be viewed in additional file 1). This was followed by the data collection material. The data collection material consisted of an interview schedule, Talking Mats™ visual framework material together with visual and receptive language screening tool. The development process is elaborated upon in the subsequent sections.

#### Development of the ICF activity and participation domain exemplars

In working with this population it was decided to depict the domain together with two examples from the domain in an attempt to ensure that participants understood what the domain represented. The identification of the two graphic symbols which were used as exemplars proceeded as follows. Two groups of students (n = 30) were asked to rate the three symbols they felt were the most representative symbols of each domain. The one group had both the graphic symbols and the words and the other group only had the symbols. The two lists were merged and the most frequently occurring symbols on both lists were developed into a composite list. Thereafter rehabilitation professionals (n = 10) were asked to place the symbols on this composite list into the correct domains. The three items which were most consistently placed in the domain by most professionals were then selected to represent each domain during testing (see additional file 1 for the domains and exemplars).

#### Construction of the Talking Mats™ and symbols

In the Talking Mats™ goal-setting package, items from the ICF relating to the nine activities and participation domains are depicted in graphic format (using picture communication symbols) so that individuals with communication difficulties may participate in the goal-setting process [13]. Picture communication symbols are commercially available line drawings which are widely utilized to supplement verbal expression for individuals with little or no functional speech. In the current study these symbols were printed on 5 cm × 5 cm cardboard, cut out and laminated and velcro was attached to the back for easy placement on a regular short pile mat. These line drawings could then be placed by the participants on the mat under the visual representation of the Likert scale which was used to indicate the level of importance participants assigned to a particular domain within the current rehabilitation program.

#### Development of the Talking Mats™ interview schedule

An interview schedule was compiled to ensure consistent presentation to each participant. The interview schedule provided a description of each of the 9 ICF activities and participation domains, as well as the question which asked the participant to rate the importance of each of the nine domains. A three-point Likert scale was constructed for use in the study, and pictorial representations for each of the options (*very important, less important*, and *not important at all) *were developed. A series of three trial items were developed, and included in the interview schedule in order to ensure that the participants understood the instructions and were able to complete the task that was required of them. The wording of the interview schedule for both groups of participants (professionals and clients) was essentially the same, and covered exactly the same content. However, due to the fact that the professionals themselves did not display any motor, cognitive, or communication difficulties, they were not required to complete either the screening protocol or the trial items from the interview schedule.

#### Development of the visual and receptive language screening tool

The aim of the screening tool was to ensure that participants would have the language and visual abilities needed in order to complete the adapted interview schedule. For the visual discrimination task, a grid with six PCS line drawings was compiled. In the screening protocol it was assumed that the participant would be able to place a card with a specific line drawing onto the matching line drawing within the grid. In order to continue with the main study, it was agreed upon that the participant would have to complete the visual discrimination task and correctly match a minimum of four out of the six items.

Five grids, each consisting of six PCS line drawings were compiled for the receptive language task. It was assumed that the participants would be able to point to the line drawing representing a specific verbal concept on request. To continue with the main study, it was agreed upon that participants had to obtain an accuracy score of at least sixty percent (correctly identify three out of the five concepts). During this test the researchers would also be able note what modifications would needed in order to accommodate the client's level of motor functioning during completion of the interview schedule.

### Pilot testing the data collection procedure and material

The aim of the pilot study was to determine the effectiveness of the screening materials and the interview schedule and (Talking Mats™ and the line drawings). The material and procedural efficacy was also assessed and modified as needed. Five clients (three with a stroke and two with traumatic brain injury) as well as three occupational therapists and one physiotherapist participated. All the participants met the selection criteria proposed for the main study. Minor adaptations to the instructions and procedure of the interview were made following the pilot study.

### Data collection

Once the rehabilitation clients gave their consent to take part in the study the screening tool was administered. If the participant successfully completed the screening and trial items, the interview was completed using the Talking Mats™ visual framework activity, according to the predetermined interview script. After completion of the Talking Mats™ the researchers checked that the client was satisfied with their choices and then captured a visual image of the completed mat with a digital camera. After all the clients completed the screening and Talking Mats™ activity, the same procedure was followed with the professionals working with these clients, excluding the administration of the screening protocol and trial items. The professionals completed a separate interview and corresponding Talking Mats™ for each of the clients to whom they provided rehabilitation services.

All participants (clients and professionals) were called into a room with minimal distractions. One of the researchers conducted the interview and was seated opposite the participant at the table. Another researcher operated the video camera and took a digital photograph of each participant's mat after it had been completed. This visual representation then formed the basis for the analysis of the data. Responses were converted into a raw score which were captured in an Excel spreadsheet. This was then analysed using relevant statistical procedures.

### Data Analysis

The video recordings of thirty percent of the completed interviews were randomly selected and checked for procedural integrity by a B. Social Science (Hons) student, specializing in Psychology. An overall rating of ninety-one percent was obtained which indicates a high level of procedural integrity across the selected interviews.

Once this had been established the mean and standard deviation [17] were computed across each of the nine domains in order to establish the importance of each of the nine ICF domains according to each participant group. For the purpose of data analysis results obtained from each of the participant groups was analysed individually. Thereafter a Friedman analysis [19] was used to explore the difference in ratings of importance across the nine ICF activities and participation domains. The Friedman analysis is a non-parametric statistical test which is an alternative to the repeated analysis of variance measure. Statistical significance is reported at the ten percent level, and the results from the Friedman analysis appear in Table 1.

**Table 1:** Ratings of the 9 ICF activities and participation domains across participant groups (differentiation based on Whiteneck and Dijkers [20])

ICF domains		Participants										
		
		Clients (n = 12)		SLT (n = 12)		OT (n = 11)		PHY (n = 11)		SW (n = 11)		p-value
		
		Mean	SD	Mean	SD	Mean		Mean	SD	Mean	SD	
Activities	Learning and Thinking	2.42	0.79	2.25^ab^	0.87	2.55	0.52	2.09^abc^	0.94	2.64	0.67	0.5612
	
	Coping	2.58	0.67	2.92^ab^	0.29	2.73	0.65	2.55^abc^	0.69	2.73	0.47	0.7688
	
	Communication	2.58	0.67	3.00^b^	0.00	2.73	0.65	2.91^bc^	0.30	2.73	0.65	0.6590
	
	Mobility	2.67	0.50	3.00^b^	0.00	2.91	0.30	3.00^c^	0.00	3.00	0.00	0.5578
	
	Self-care	2.75	0.62	2.92^ab^	0.29	2.82	0.60	2.82^abc^	0.60	2.91	0.30	0.9513
	
	Domestic Life	2.30	0.78	2.25^ab^	0.62	2.36	0.81	1.91^abc^	0.83	2.45	0.52	0.6990

Participation	Relationships	2.50	0.80	3.00^b^	0.00	2.82	0.40	2.64^abc^	0.67	2.82	0.40	0.7907
	
	Work and Education	2.75	0.62	2.58^ab^	0.67	2.73	0.47	1.91^abc^	0.70	2.18	0.98	0.1861
	
	Spare Time	2.42	0.67	2.00^a^	0.74	2.18	0.60	1.82^a^	0.75	2.36	0.81	0.5612

	p-value	0.7857		0.0002*		0.3078		0.0007*		0.4502		

Differing postscripts indicate a significant difference at the 10% level

Abbreviations: SLT: speech-language therapist; OT: Occupational therapist; PHY: physiotherapist, SW: social worker.

## Results and Discussion

The main aim of this study was to describe the importance ratings assigned to each of the ICF activities and participation domains by rehabilitation professionals and clients in an acute rehabilitation context utilizing the Talking Mats™ visual framework. The results will be discussed in terms of the rehabilitation priorities (those domains which were rated by a group of participants as being very important for inclusion in the current rehabilitation program). Similarities and differences between the participants groups will be explored. Importance ratings, or priorities, are described according to the mean score each group assigned to each of the nine ICF domains. Maximum scores that could be obtained for each ICF domain by each participant was 3 (indicating very important) and the minimum score was 1 (indicating not at all important). This section will conclude with a discussion regarding the relevance of using the Talking Mats™ visual framework for this population and within an acute care context.

### Ratings from each participant group indicating the importance of including each ICF domain in the current rehabilitation program

The mean scores for each of the nine different domains ranged from 1.82 (0.75) to 3.00 (0.00) and can be viewed in Table 1. High means (a score of 2.5 and above) indicated that the participants felt that this domain was a priority in the current rehabilitation program.

All participant groups (clients and professionals) rated coping, communication, mobility, self-care, and relationships as important domains for inclusion in the rehabilitation program, as can be viewed in Table 1. In addition, all the participant groups rated spare time and domestic life, as less important for inclusion in the acute-care rehabilitation program. The remaining two domains (namely leaning and thinking and work and education) show the greatest variation in the ratings across the participant groups. The means for learning and thinking domain ranges from 2.09 (0.94) to 2.64 (0.67) whilst the mean values for work and education domain ranged from 1.91 (0.7) to 2.75 (0.62).

The results indicate a high degree of consensus in terms of those domains which participants' rated as important in the current rehabilitation program. The domains which are consistently rated as most important (i.e. communication, mobility and self-care) appear to be primarily activity domains. Results from a study which used the ICF to identify priorities between professionals, parents and youth with cerebral palsy also indicated mobility and communication amongst the top priorities across all the participants [16]. The domains highlighted as important in both these studies appears to be similar, with the exception of the current participants rating of the relationships domain, which is often viewed as a participation domain [20].

A Friedman analysis was then conducted; however no statistically significant differences were reported for the ratings across the participant groups. This supports the finding that priorities and non-priorities can be highlighted and agreed upon by all the participant groups who took part in this study. It is therefore feasible to deduce that consensus is indeed possible between clients and professionals within the acute-care setting. It also implies that professionals in the rehabilitation team are able to identify domains which were not primarily within their scope of practice as important domains (speech-language therapists identified mobility and self-care as important while physiotherapists identified communication as important).

### The differences in importance ratings for each of the ICF domains within each of the professional participant groups

Each group of professional's responses were then compared across all of the clients that they treated to determine if any patterns exist in terms of the importance certain professional disciplines placed on certain domains. Results from this Friedman analysis indicated statistically significant differences between domain priorities exist in two professional participant groups, namely the speech-language therapists and the physiotherapists. Results can be viewed in Table 1.

For both of these groups the differences are between the spare time domain and the domains of communication and mobility. For the speech-language therapists this difference is also present between spare time and relationships. This trend seems to indicate that the domain of spare time is not considered as important to include as a rehabilitation priority in the acute-care setting by certain professionals. However if the activity and participation domains are indeed conceptually different as proposed by Whiteneck and Dijkers [20] and Badley [21] it would imply that professionals and clients intuitively distinguish between domains which are more activity focussed and those which are more focussed on participation. If this distinction is upheld then communication and mobility are defined as predominantly assessing functioning at an activity level (which is focussed at the level of the individual) and spare time is predominantly assessing participation aspect of this component (focussed at a social level). Therefore at the acute stage of rehabilitation professionals and clients themselves appear to focus on addressing activity limitations and place less importance on addressing possible participation restrictions.

### Relevance of using the Talking Mats™visual communication framework within an acute-care setting

The use of the Talking Mats™ visual communication framework therefore appears to be an effective manner of communicating with people who have an acquired communication disorder in order to determine rehabilitation priorities within the acute stage of rehabilitation. This may possibly be due to the fact that the framework uses symbols to augment the verbal and written input traditionally given during activities such as goal-setting. The degree of overlap between professionals and clients regarding the important domains for inclusion indicate that this is a reliable method for assisting individuals with an acquired communication disability to participate in the identification of rehabilitation priorities. It appeared as if Talking Mats™ provided the clients the opportunity to express their views, which they would have had difficulty doing using a more traditional interview format. Murphy [22] reported similar results when using the Talking Mats™ visual communication framework on frail-older adults with a range of communication disabilities. She concludes that Talking Mats™is a strategy that not only allows frail-older people the opportunity to voice their opinion, but also provides a way for their input to be captured and shared with other interested parties [22].

### Strengths, Limitations and Areas for Further Research

This study has several strengths and limitations which will be discussed below. Due to the small number of participants (12 clients and 20 professionals) together with the fact that the main data collection occurred at a single site, means that the results must be generalized with caution. A further study with a larger number of participants would serve to further support the results obtained from this group of participants.

Talking Mats™ is however, a well researched framework that enables people with acquired communication difficulties to actively participate in setting rehabilitation goals. This study provides initial evidence to suggest that the Talking Mats ™ visual communication framework is also appropriate for use within the acute-rehabilitation context. Results indicated that clients' priorities were in fact similar to those identified by the rehabilitation professionals, when using this framework. Professionals and clients are therefore, to a large extent, able to agree on the rehabilitation priorities within an acute-care setting. Four important domains were highlighted by both groups. These domains dealt primarily with activity limitations pertaining to mobility and communication. This information can be used to set rehabilitation goals which both the client and professional feel are meaningful. This is in keeping with a client-centred approach as it enables greater client self-determination and control, which enhances the potential for active and sustained participation within the rehabilitation program [23]. Although this study investigated the usefulness of the ICF activity and participation at a domain level to identify broad areas for inclusion in rehabilitation, further research is needed to highlight client priorities regarding specific aspects within one or two of these domains e.g. communication or mobility.

It is important to note that while Talking Mats ™ provides a simple framework to obtain clients' opinions; this methodology does possess a few constraints. Firstly, a considerable amount of planning is involved in the preparation of the material for use in the activity as indicated by Murphy [22]. Furthermore, due to the fact that this activity involves placing a symbol on the mat, a certain level of motor, visual and language ability are required in order to participate in this activity. It is therefore clear that an adapted Talking Mats ™ interview may not be a suitable tool to assist in identifying rehabilitation priorities with all clients. In this current study three of the participants were unable to complete the activity due to limitation in their receptive language abilities. Another constraint is that the views captured on the Talking Mats ™represent a participant's current opinions. An additional avenue of research might therefore be to investigate the changes in rehabilitation priorities over time and compare the level of agreement between professionals and clients as rehabilitation progresses.

A final limitation of the current study is that there were a limited number of professionals who were involved in providing rehabilitation services to the clients. As a result the same professional provided rehabilitation to more than one client. This may have negatively impacted on the ability to focus on the priorities for a specific client, as professionals were required to complete an interview and Talking Mats ™ for each client they provided services to.

## Conclusions

Person-centred care focuses on the importance of cultivating the client as an active member of the rehabilitation team. When using the ICF's nine activities and participation domains as a common language it is possible to identify important domains to prioritize in the rehabilitation program. Findings from this study indicate that using a Talking Mats™ activity for individuals with an acquired communication disability, allows for these clients to participate in a meaningful way in the process of identifying important domains to be addressed during rehabilitation goal-setting. When comparing results between professionals and clients in an acute-care rehabilitation program, it is evident that a certain degree of agreement regarding both the domains of importance and non-importance is indeed possible. The majority of the domains which are viewed as important for inclusion at this stage of the rehabilitation process can be classified as activity domains (learning and thinking, communication, mobility and self-care). This would imply that the focus during the acute stage of rehabilitation is on addressing activity limitations as opposed to participation restrictions. When clients are able to reliably identify priorities and actively participate in establishing priorities for their own rehabilitation program it facilitates an increase in their sense of control and independence, which aligns with the tenets of a person-centred approach to rehabilitation.

## Competing interests

The authors declare that they have no competing interests.

## Authors' contributions

MH conceptualized the study, and participated in its design, statistical analysis and drafted the manuscript. MG and AVDM participated in its design, data collection and statistical analysis. All authors read and approved the final manuscript.

## Supplementary Material

Additional file 1 **Talking Mats™ symbols of the nine ICF domains and their exemplars**. Click here for file
