# Supplementary material for: The ICF as a common language for rehabilitation goal-setting: comparing client and professional priorities
Source: Health Qual Life Outcomes. 2011 Oct 7;9:87. doi: 10.1186/1477-7525-9-87 (PMC3204224; doi:10.1186/1477-7525-9-87)
Supplement: Additional file 1 — Talking Mats™ symbols of the nine ICF domains and their exemplars. [file 1477-7525-9-87-S1.DOCX]

**Talking Mats™ symbols: nine ICF domains and their exemplars**

| **Chapter number** | **Domains** | **Exemplars** |
| --- | --- | --- |
| D1 | 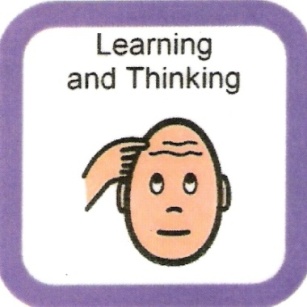 | 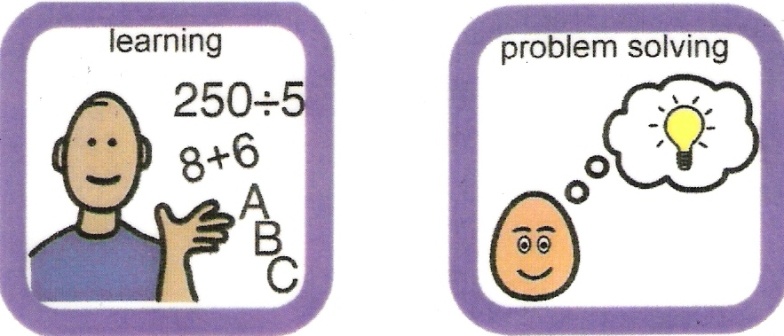 |
| D2 | 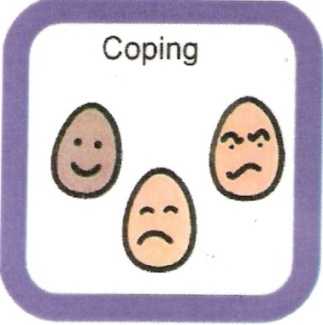 | 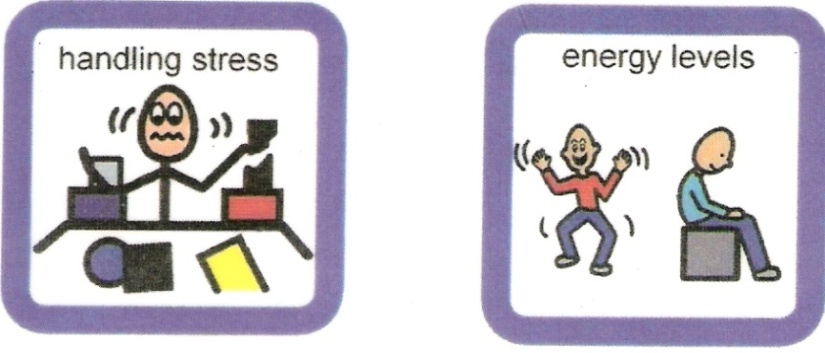 |
| D3 | 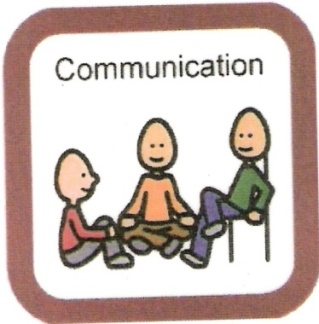 | 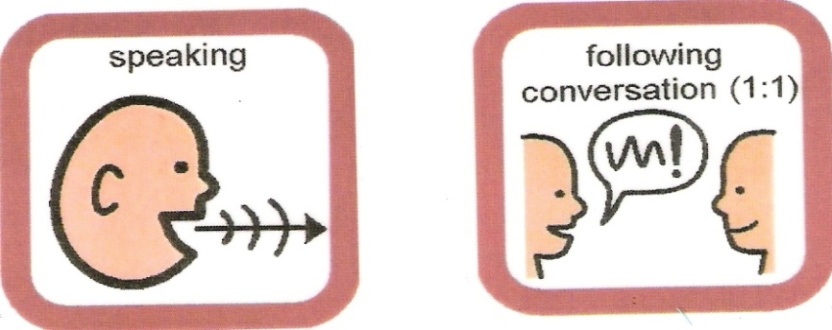 |
| D4 | 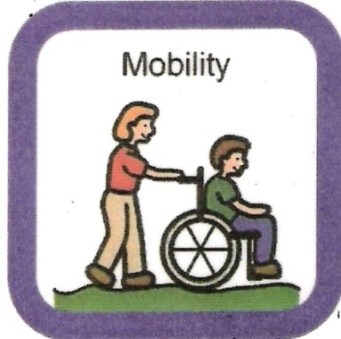 | 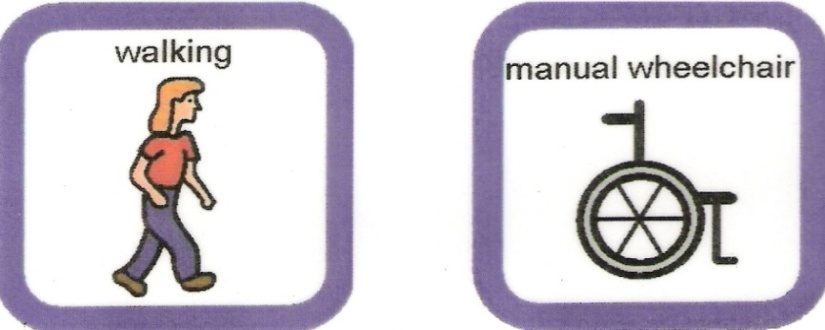 |
| D5 | 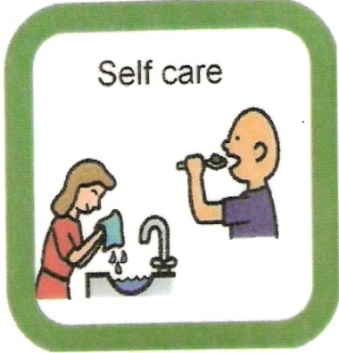 | 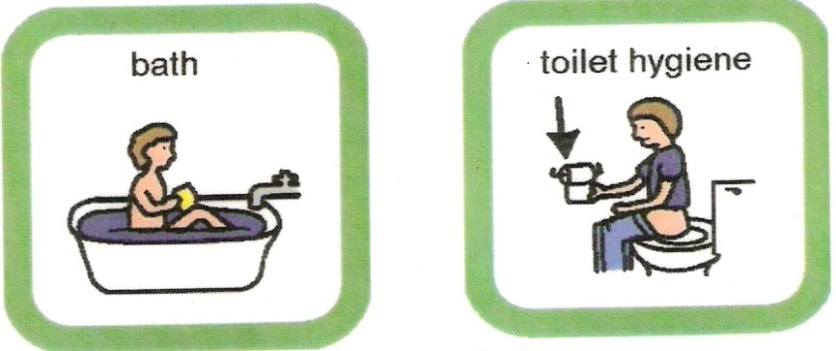 |
| D6 | 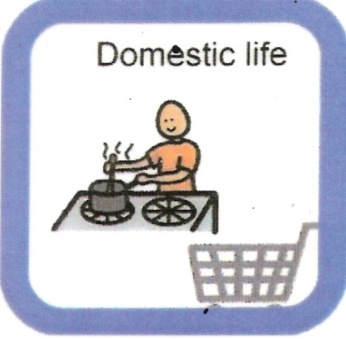 | 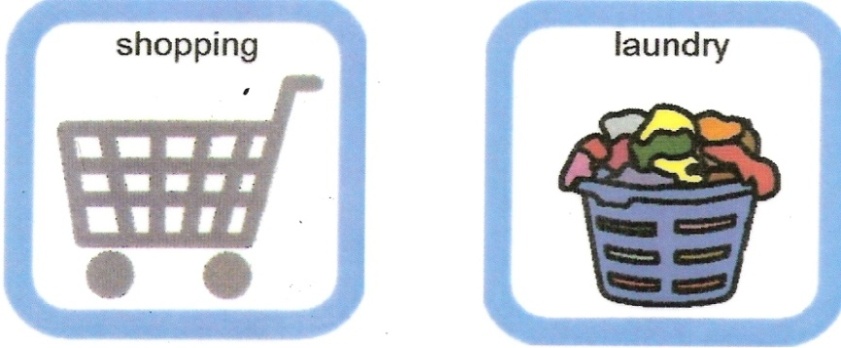 |
| D7 | 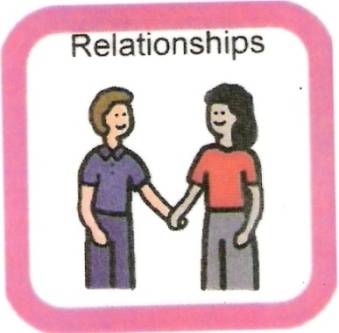 | 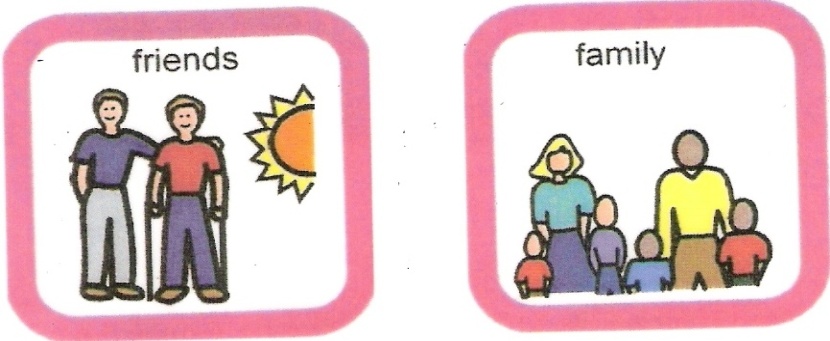 |
| D8 | 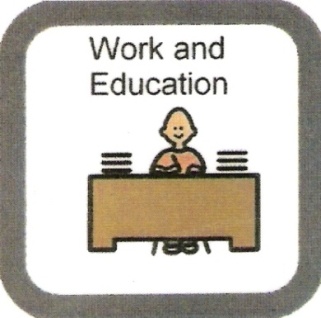 | 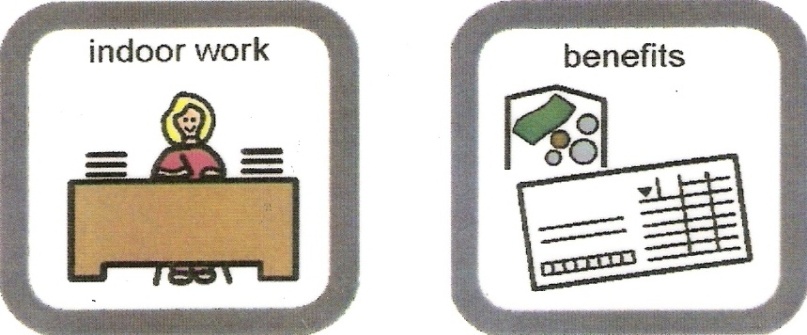 |
| D9 | 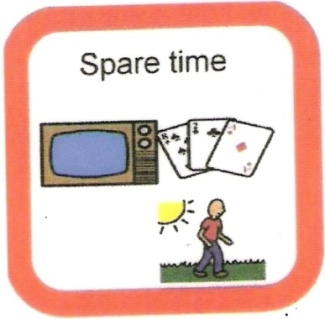 | 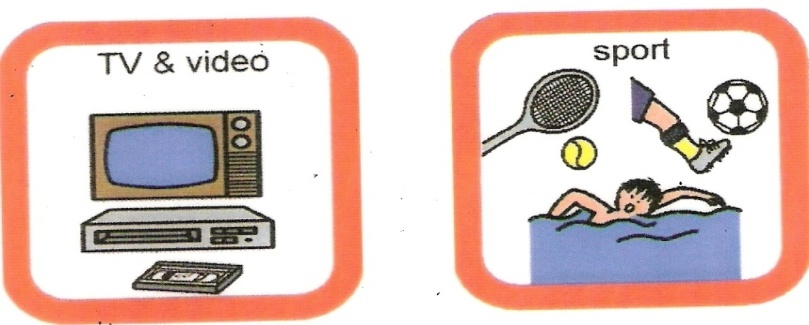 |
